# Can STEreotactic Body Radiation Therapy (SBRT) Improve the Prognosis of Unresectable Locally Advanced Pancreatic Cancer? Long-Term Clinical Outcomes, Toxicity and Prognostic Factors on 142 Patients (STEP Study)

**DOI:** 10.3390/curroncol30070513

**Published:** 2023-07-24

**Authors:** Tiziana Comito, Maria Massaro, Maria Ausilia Teriaca, Ciro Franzese, Davide Franceschini, Pierina Navarria, Elena Clerici, Luciana Di Cristina, Anna Bertolini, Stefano Tomatis, Giacomo Reggiori, Andrea Bresolin, Silvia Bozzarelli, Lorenza Rimassa, Cristiana Bonifacio, Silvia Carrara, Armando Santoro, Alessandro Zerbi, Marta Scorsetti

**Affiliations:** 1 Radiotherapy and Radiosurgery Department, Humanitas Cancer Center, IRCCS Humanitas Research Hospital, 20089 Milan, Italy; tiziana.comito@cancercenter.humanitas.it (T.C.); maria.ausilia.teriaca@cancercenter.humanitas.it (M.A.T.); ciro.franzese@hunimed.eu (C.F.); davide.franceschini@cancercenter.humanitas.it (D.F.); pierina.navarria@cancercenter.humanitas.it (P.N.); elena.clerici@cancercenter.humanitas.it (E.C.); luciana.dicristina@cancercenter.humanitas.it (L.D.C.); anna.bertolini@cancercenter.humanitas.it (A.B.); stefano.tomatis@cancercenter.humanitas.it (S.T.); giacomo.reggiori@cancercenter.humanitas.it (G.R.); andrea.bresolin@cancercenter.humanitas.it (A.B.); marta.scorsetti@hunimed.eu (M.S.); 2 Department of Biomedical Sciences, Humanitas University, 20072 Milan, Italy; lorenza.rimassa@hunimed.eu (L.R.); armando.santoro@hunimed.eu (A.S.); alessandro.zerbi@hunimed.eu (A.Z.); 3 Medical Oncology and Hematology Unit, Humanitas Cancer Center, IRCCS Humanitas Research Hospital, 20089 Milan, Italy; silvia.bozzarelli@cancercenter.humanitas.it; 4 Department of Radiology, Humanitas Cancer Center, IRCCS Humanitas Research Hospital, 20089 Milan, Italy; cristiana.bonifacio@humanitas.it; 5 Department of Gastroenterology, Humanitas Cancer Center, IRCCS Humanitas Research Hospital, 20089 Milan, Italy; silvia.carrara@humanitas.it; 6 Department of Pancreatic Surgery, Humanitas Cancer Center, IRCCS Humanitas Research Hospital, 20089 Milan, Italy

**Keywords:** radiotherapy, locally advanced pancreatic cancer, unresectable pancreatic cancer, stereotactic body radiation therapy

## Abstract

Aim: The gold standard of care for pancreatic adenocarcinoma is the integrated treatment of surgery and chemotherapy (ChT), but about 50% of patients present with unresectable disease. Our study evaluated the efficacy in terms of local control, survival and safety of stereotactic body radiation therapy (SBRT) in locally advanced pancreatic cancer (LAPC). Methods: A retrospective study (STEP study) analyzed patients with LAPC treated with a dose of 45 Gy in 6 fractions. Local control (LC), distant progression free survival (DPFS), overall survival (OS) and toxicity were analyzed according to the Kaplan-Meier method. Results: A total of 142 patients were evaluated. Seventy-six patients (53.5%) received induction ChT before SBRT. The median follow-up was 11 months. One-, 2- and 3-year LC rate was 81.9%, 69.1% and 58.5%. Median DPFS was 6.03 months; 1- and 2-year DPFS rate was 19.9% and 4.5%. Median OS was 11.6 months and 1-, 2- and 3-year OS rates were 45.4%, 16.1%, and 9.8%. At univariate analysis, performed by the log-rank test, age < 70 years (*p* = 0.037), pre-SBRT ChT (*p* = 0.004) and post-SBRT ChT (*p* = 0.019) were associated with better OS. No patients experienced G3 toxicity. Conclusion: SBRT represents an effective and safe therapeutic option in the multimodal treatment of patients with LAPC in terms of increased LC. When SBRT was sequentially integrated with ChT, the treatment proved to be promising in terms of OS as well.

## 1. Introduction

Pancreatic cancer (PC) has a poor prognosis, with a 5-year overall survival (OS) rate of about 10% [1]. Currently, PC represents the fourth leading cause of cancer-related deaths in Western countries [1,2] and it is expected to become the second leading cause of cancer-related deaths by 2030 [3].

Although surgery is the only potentially curative treatment, more than 50% of patients are diagnosed with unresectable PC caused by vascular involvement without distant metastasis, which is defined as locally advanced pancreatic cancer (LAPC) [4].

Pancreatic ductal adenocarcinoma (PDAC) is typically considered a systemic disease, as its natural history is dominated by the development of metastases. Therefore, chemotherapy (ChT) is the standard of care for LAPC, according to current guidelines [5]. At the same time, local progression significantly contributes to morbidity and mortality. Therefore, about a third of patients with PC die from complications related to local tumor progression in the absence of metastases, according to an analysis of autopsy series by Johns Hopkins Medical Institution [6]. Furthermore, Crane et al. showed that local tumor progression is the most common cause of death in patients alive after more than 16 months from diagnosis [7].

According to this evidence, the integration of chemotherapy and radiation treatment could represent a valid therapeutic option in the management of LAPC. Nevertheless, some retrospective studies have suggested that induction chemotherapy administered before concurrent chemoradiotherapy could improve the survival of patients with LAPC [8,9].

Conventional fractionated radiation therapy (CFRT) has been shown to improve local pain control and to maintain the stability of disease progression. Nevertheless, these clinical benefits of ChT-RT in terms of local pain control have been limited by the long courses of therapy, the significant toxicity caused by a lack of dose homogeneity and the concurrent approach, and the decreasing use of full-dose systemic therapy with ChT-RT [10].

In the last few years, stereotactic body radiation therapy (SBRT) has emerged as a viable alternative to ChT-RT in the treatment of LAPC. SBRT is a radiation technique that allows the delivery of an ablative dose to the target lesion and a minimal dose to adjacent critical structures in a short treatment time, with higher local control and an optimal toxicity profile, without affecting the sequential integration of full-dose systemic therapy.

However, the optimal radiation treatment modality and, particularly, the optimal radiation treatment timing for LAPC remain debated.

Among the reasons for these open questions, we should include the characteristics of the studies currently available. Therefore, the literature about SBRT in unresectable PC encompasses series composed of a limited number of patients treated with nonhomogeneous prescription RT doses, fractionations and chemotherapy regimens. According to major prospective and retrospective studies published between 2013 and 2021 (Table 1 and Table 2), which analyzed an average of 40 patients (range 11–63 patients), the median 1y-LC was 81%, and the median OS and 1y-OS rate were 13.8 months and 59%, respectively.

Parallel to the outcome data, the first prospective studies about SBRT in LAPC, which evaluated the treatment delivered in a single fraction [25,26,27], recorded a significant gastrointestinal toxicity rate.

Thereby, in our study, we have collected and analyzed data related to the delivery of SBRT in a large number of unresectable patients treated with homogeneous dose prescription, fractionation scheme, and RT technique, reporting the long-term clinical outcomes and toxicity.

## 2. Materials and Methods

### 2.1. Study Population

Unresectable patients with LAPC were identified from a prospectively collected mono-center database started with a prospective phase 2 study, which provided the 6-fraction SBRT in PC [14].

This retrospective monocentric study was approved by the Humanitas Ethical Review Board (n° 33/22_14 June 2022).

All procedures performed were in accordance with the ethical standards of the Institution and with the 1964 Helsinki Declaration. Informed consent was obtained from all treated patients. All patients were discussed in a multidisciplinary tumor board composed of a medical oncologist, a biliary–pancreatic surgeon, a radiologist, a gastroenterologist, a pathologist and a radiation oncologist.

The inclusion criteria were:

- Age ≥ 18 years old
- Minimum Karnofsky Performance Status of 70%
- Histologically and/or radiologically proven locally advanced unresectable PC
- Tumor diameter < 5 cm
- Absence of nodal and metastatic disease

The exclusion criteria included:

- Other malignancies diagnosed within 5 years
- Previous abdominal SBRT
- Gastric or duodenal obstruction
- Nodal metastases
- Metastatic disease
- Concomitant ChT

Unresectable LAPC was defined according to the American Hepato–Pancreato–Biliary Association/Society of Surgical Oncology/Society for Surgery of the Alimentary Tract criteria [28].

### 2.2. Stereotactic Body Radiation Therapy

During the simulation phase, all patients were immobilized in a supine position with the arms above the head, using a thermoplastic body mask. A contrast-free and a triphasic contrast-enhanced computed tomography (CT) scan with a slice thickness of 3 mm was acquired for all patients. The 4-dimensional CT (4D-CT) imaging was performed in patients with respiratory excursions greater than 5 mm.

The Gross Tumor Volume (GTV) was defined by macroscopic disease. In selected patients, the simulation CT images were co-registered with the magnetic resonance images (MRI) and/or fluorodeoxyglucose-positron emission tomography (FDG-PET CT) to better identify the GTV. No elective lymph node irradiation was performed. The Clinical Target Volume (CTV), defined as GTV with no additional margins, was delineated on the venous phase of the CT scan.

In all patients who underwent a 4D-CT scan, an internal target volume (ITV) was defined as the volume of the CTV delineated by the breathing phases acquired during the 4D CT.

Another 6 mm isotropic expansion was generated from the ITV to obtain the Planning Target Volume (PTV).

The critical structures (organs at risk, OaR), including the stomach, duodenum, small bowel, kidneys, liver and spinal cord, were delineated.

Dose prescription was 45 Gy in 6 consecutive fractions (BED_10_ = 78.75 Gy) and the treatment was delivered with volumetric modulated arc therapy (VMAT) by RapidArc tecnique.

A required target coverage of D98% > 98% for the CTV. A maximum acceptable dose heterogeneity to the PTV of D98% > 95% and D2% < 107%.

Dose-volume constraints for the OaR were: spinal cord D1cc < 27 Gy, kidneys V15Gy < 30%, duodenum V36Gy < 1 cc, stomach V36Gy < 1 cc, small bowel V36Gy < 3 cc, liver (Vwhole liver–V21Gy) > 700 cc.

**Figure 1:**
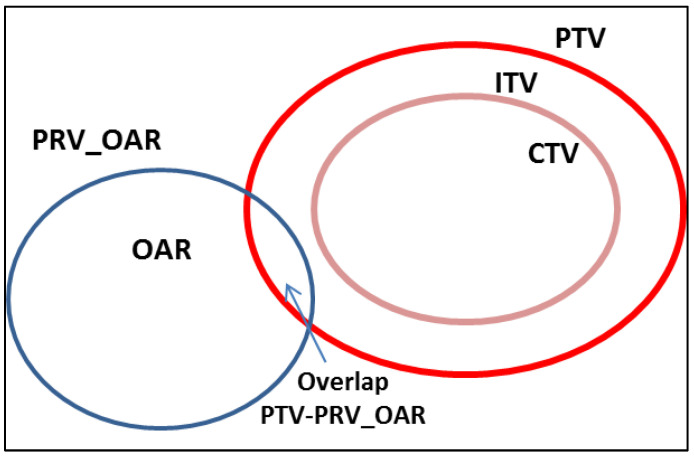
A prescription dose of 45 Gy in 6 fractions was delivered with a maximum acceptable dose heterogeneity to the PTV of D98% > 95% and D2% < 107%. In the area of overlap between PTV and OaR, we delivered the maximum tolerated dose by OaR. PRV: Planning Organs at Risk Volume; OAR: Organ at Risk; PTV = Planning Target Volume; ITV: Internal Target Volume; CTV = Clinical Target Volume.

In the area of overlap between PTV and OaR, we delivered the maximum tolerated dose by OaR to minimize the dose inhomogeneity on PTV according to dose-volume constraints, as shown in Figure 1.

To reduce interfraction variability, all patients kept a fast of at least 3 h before treatment.

Before every treatment session, the setup of patients was verified daily by image guidance with Cone Beam CT (CBCT). Regarding patients’ setup, after an initial adjustment based on bone anatomy, the setup was then adjusted to match soft tissue structures, such as main blood vessels.

### 2.3. Response Evaluation

Patients were reassessed 8 weeks after SBRT and then every 3 months. Clinical evaluation, blood exams with CEA and CA 19-9, a contrast-enhanced CT scan were performed; patients undergoing staging FDG-PET CT before SBRT have performed a re-staging FDG-PET CT 4–6 months after treatment.

Response assessment was performed according to CA19-9 levels, Response Evaluation Criteria in Solid Tumors v1.1 (RECIST) [29], and PET Response Criteria in Solid Tumors (PERCIST) [30]. According to RECIST criteria, a Complete Response (CR) was defined as the disappearance of the target lesion; a Partial Response (PR) was defined as a decrease in at least 30% in the sum of the diameter of the target lesion, taking as reference the baseline sum diameter; a Progressive Disease (PD) was defined as an increase in at least 20% in the sum of the diameter of the target lesion, taking as reference the smallest sum on the study; Stable Disease (SD) was defined as less than 30% reduction in the tumor major diameter or less than 20% increase in tumor major diameter.

**Figure 2:**
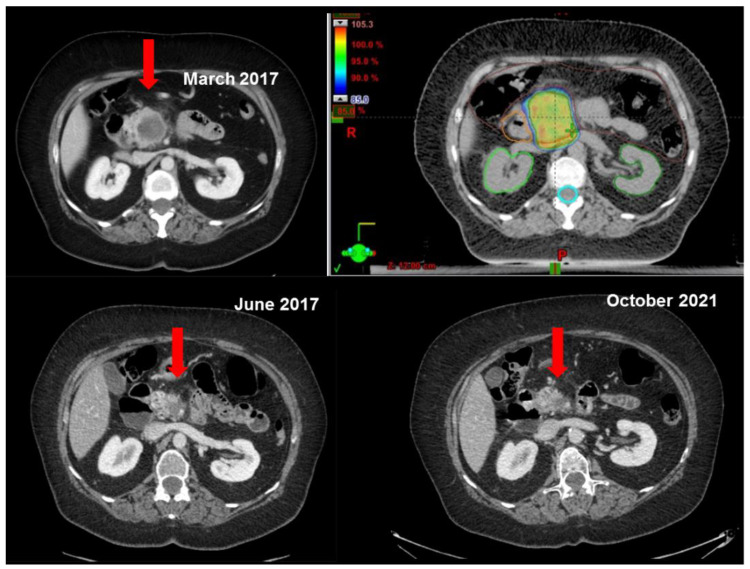
Example of dose distribution and radiological response after SBRT.

An example of dose distribution and radiological response after SBRT is reported in Figure 2.

We also evaluated the clinical response in patients presenting with pain before radiation therapy according to the Numerical Rating Scale (NRS) scoring system.

The toxicity profile was assessed according to the National Cancer Institute (NCI) Common Terminology Criteria for Adverse Events (CTCAE) v5.0 [31].

Acute toxicity was defined as toxicity described within 90 days of the end of SBRT, while late toxicity was defined as occurring beyond 90 days.

### 2.4. Statistical Analysis

We performed the statistical analysis with the software STATA 14. The primary endpoint was local control (LC), which was defined as the time interval between the end of the SBRT and the first evidence of local progression (in-field), and patients were censored from the date of death. Secondary endpoints were distant progression free survival (DPFS), overall survival (OS), and toxicity profile. DPFS was defined as the time interval between the end of SBRT and the first finding of a distant progression (out-of-field). Overall survival (OS) was calculated from the end of the SBRT to the patient’s death or the last follow-up.

Local control, DPFS and OS were calculated according to the Kaplan-Meier method; the log-rank test was used for the univariate analysis to evaluate a correlation with prognostic factors such as age, tumor diameter, CTV, PTV, time between diagnosis and SBRT, ChT before SBRT and ChT after SBRT, and pre-SBRT and post-SBRT CA 19-9 values. A multivariable analysis of the endpoints with prognostic factors such as age, ChT before SBRT and ChT after SBRT was performed by Cox regression. A *p* value < 0.05 was considered statistically significant.

## 3. Results

Between January 2011 and June 2021, 142 patients with LAPC were treated in a single institution. Demographic, clinical, and treatment characteristics are shown in Table 3.

The median age was 71 years (41–91). Sixty-three (44%) patients were younger than 70 years and 79 (66%) patients were 70 years, of age or older than 70 years.

The histological diagnosis was not available in 15 patients (10.5%), who were very elderly and frail and not candidates for chemotherapy.

In 124 patients (87.3%), the CA 19.9 value was available before SBRT, with a median range of 102.75 U/mL (0.8–12.000). In 79 patients (63.7%), the CA 19.9 value was <300 U/mL, in 45 patients (36.3%), it was ≥300 U/mL, and their changes were not statistically significant.

Most of the tumors were located in the head (91 lesions, 64.1%), then in uncinate process (23 lesions, 16.2%), then in body (20 lesions,14.1%) and the others were located in tail (3, 2,1%) and isthmus (5, 3.5%).

Seventy-six patients (53.5%) underwent induction ChT that ended at least 2 weeks prior to SBRT. The number of ChT cycles ranged from 3 to 10. This variability was related to performance status (PS), age, comorbidities, patient compliance, tumor response, and toxicity. In 55 patients (72%), gemcitabine-based ChT was administered: 7 gemcitabine monotherapy, 21 gemcitabine oxaliplatin (GEMOX), 10 PEX-G (cisplatin, epirubicin, 5-fluorouracil, gemcitabine), 17 gemcitabine-nab paclitaxel, whereas 18 patients (23.7%) received folinic acid, 5-fluorouracil, irinotecan and oxaliplatin (FOLFIRINOX). In 42 patients (29.6%), ChT was also administered after SBRT. The median follow-up (mfup) was 11 months (range: 6–55 months). No patient had to discontinue radiation treatment.

Twelve patients (8.4%) were alive at the time of the analysis, and seven of them (5%) had no evidence of local or metastatic disease. After SBRT, four patients (3%) underwent surgery with negative margins.

Based on the NRS scoring system, 45 (31.6%) patients presented with pain before SBRT. In 14 (9.8%) patients, pain control after radiation therapy allowed the suspension of analgesics treatment; in 10 (7.04%) patients, analgesics were reduced.

### 3.1. Local Control

**Figure 3:**
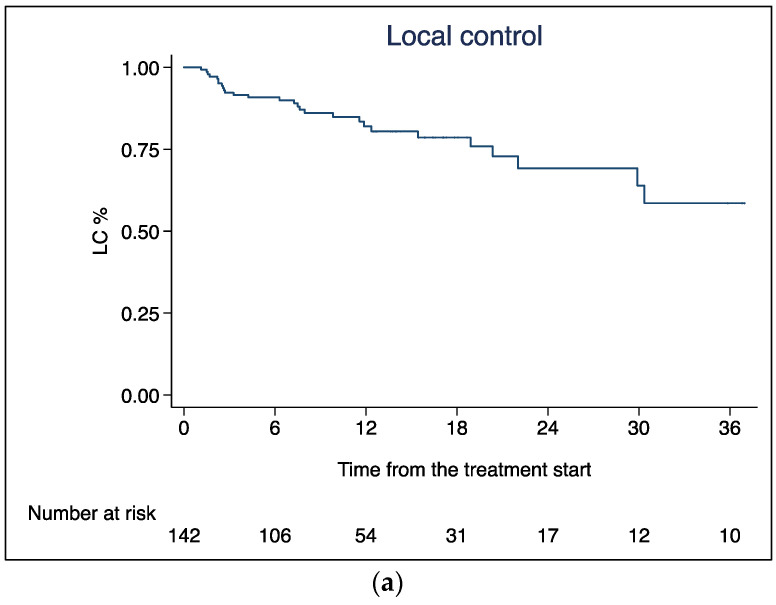

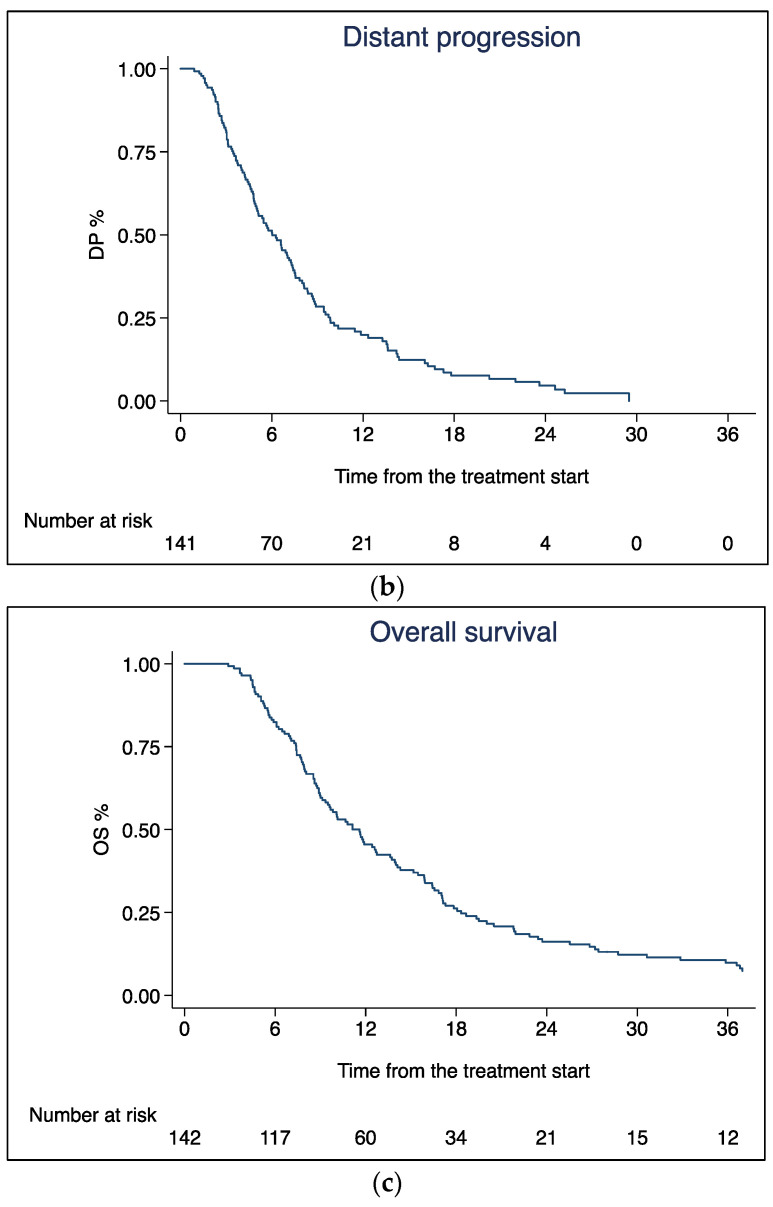
(**a**) Local control; (**b**) distant progression free survival; and (**c**) overall survival.

One-, 2-, and 3-year LC rates were 81.9% (95% CI 73.1–88.1), 69.1% (95% CI 54.7–79.8), and 58.5% (95% CI 39.5–73.4), respectively (Figure 3a). The median LC was not reached. According to RECIST criteria (Table 4), we observed one patient with a complete response (0.7%), 31 partial response cases (22%), 81 patients showed disease stability (57%), and 29 (20.3%) had a local progression. Pre-SBRT and post-SBRT FDG-PET CT were performed in 17 patients (12%); in three patients (2.1%) with stable disease on CT scan, FDG-PET CT showed a partial metabolic response, while in five patients (3.5%), with an uncertain persistence of disease on CT scan, FDG-PET CT reported a complete metabolic response, which was also confirmed at subsequent restaging CT scans. Among patients with local progression, seven patients (24.1%) presented isolated local progression of disease without distant metastases, and they underwent retreatment after a gap of one to three years from the end of SBRT. The radiation dose delivered was 20 or 25 or 35 Gy in five fractions, and RT was well tolerated in all retreated patients.

### 3.2. Distant Progression Free Survival

The median DPFS was 6.03 months. Eighty-four patients (59.1%) developed distant metastases. The sites of progression were the liver (31, 37%), peritoneum (13, 15.5%), lung (8, 9.5%), lymph nodes (4, 4.7%), bone (2, 2.3%) and other multiple sites (26, 31%). One- and 2-year DPFS rates were 19.9% (95% CI 13.4–27.2) and 4.5% (95% CI 1.6–9.7%), respectively (Figure 3b).

At progression, 42 patients (29.6%) underwent ChT. In cases of oligoprogression, RT was performed on the site of metastatic disease (14 patients, 10%): 10 patients underwent SBRT for liver metastasis (4, 3%), lung lesions (4, 3%) and lymph nodes (2, 1%). Four patients (3%) underwent symptomatic RT treatment for bone metastases.

### 3.3. Overall Survival

**Figure 4:**
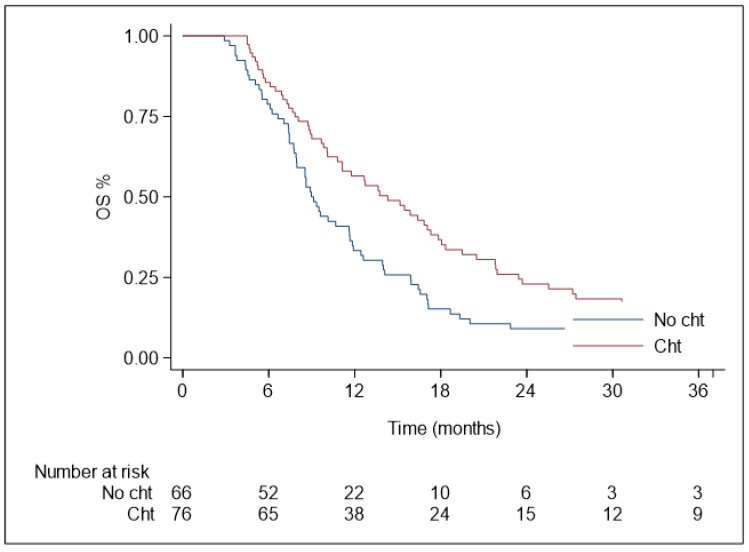
Overall survival in patients treated with chemotherapy and SBRT (n = 76) and in patients treated with SBRT alone (n = 66). Specifically, pre-SBRT ChT (HR 0.59, 95% CI 0.41–0.84, *p* = 0.004) and post-SBRT ChT (HR 0.63, 95% CI 0.43–0.92, *p* = 0.019) were significantly associated with OS.

Median OS (mOS) was 11.6 months, and the 1-, 2- and 3-year OS rates were 45.4% (95% CI 37.0–53.5), 16.1% (95% CI 10.5–22.9), and 9.8% (95% CI 5.4–15.7), respectively (Figure 3c). At the univariate analysis (Table 5), age < 70 years (HR 1.46, 95% CI 1.02-2.08, *p* = 0.037), pre-SBRT ChT (HR 0.59, 95% CI 0.41–0.84, *p* = 0.004) and post-SBRT ChT (HR 0.63, 95% CI 0.43–0.92, *p* = 0.019) were significantly associated with OS. Specifically, mOS in patients treated with induction ChT was 14.3 months, compared to 8.9 months in patients treated with exclusive SBRT (Figure 4).

In multivariable analysis, we did not find statistically significant results. (Table S1, Supplementary Files).

Before SBRT, 124 patients (87.3%) presented higher CA 19-9 values, and the analysis was repeated 8 weeks after the end of SBRT. We found stability of CA 19-9 levels in 57 patients (46%), a reduction in 49 patients (39.5%), of whom 15 patients (12%) had a decline to normal values, and an increase in marker levels in 18 patients (14.5%) who have presented local or systemic progression of disease.

### 3.4. Toxicity

Sixty-six patients (46.5%) experienced mild or moderate acute toxicity: nausea G1 in 23 patients (35%) and G2 in 14 patients (21.3%). Fifteen patients (22.7%) have reported epigastric pain (G1-G2), treated with minor analgesic drugs. There was no acute and/or late G3 toxicity.

Three patients developed ulceration of the duodenal mucosa (G2) after 8, 13, and 21 months of SBRT. In two of these patients, we observed a concurrent pathological infiltration from primary pancreatic disease.

## 4. Discussion

The role of SBRT in the treatment of unresectable pancreatic cancer was investigated and introduced in the therapeutic flowchart as a promising treatment option [5,32,33].

Nevertheless, most of these experiences have been performed on small cohorts of patients with heterogeneous characteristics, in terms of clinical features, fractionation RT schemes, and chemotherapy regimens.

The first prospective studies about SBRT in LAPC evaluated the feasibility of the single fraction [25,26,27]. A promising local control of disease was achieved at the price of significant gastrointestinal toxicity and poor overall survival, so the current studies are directed to a fractionated RT scheme in order to maintain local control and reduce the risk of toxicity to adjacent normal tissues.

Currently, there is not a consensus about the best dose-for-fraction regimen; the available literature recommends the scheme of 5 fractions with a minimum total dose of 33 Gy [13,18,33,34], 35 Gy [35], and 40 Gy [15,36,37,38,39].

In our analysis, SBRT at a total dose of 45 Gy delivered in 6 fractions showed an effective local control rate of 69.1% at two years. Considering the mOS of 14.3 months when SBRT was sequentially integrated with ChT, our results suggested that about 70% of patients treated with SBRT will be able to spend their remaining lives without relevant symptoms related to both local disease progression, such as pain, jaundice, sick and difficult duodenal transit, or radiotherapy related toxicity. All patients, indeed, tolerated radiation therapy without several complications.

Considering the clinical outcomes and toxicity profiles of the main prospective and retrospective studies published between 2013 and 2021 (Table 1 and Table 2), an average of 40 patients (range 11–63 patients) were enrolled in the study, and the most commonly used fractionation regimen was that of 5 fractions with a median total dose of 37.5 Gy. The median 1 y-LC is comparable to that obtained in our analysis (81% vs 81.9%). The median-OS and 1 y-OS rate were lower in our study (13.8 months vs. 11.6 months and 59% vs. 45.4%, respectively). This disparity may refer to the unfavorable selection of patients for pancreatic SBRT. In our study, we analyzed a large cohort of 142 patients with unresectable LAPC, and 79 of them were elderly, with an average age ≥ 70 years. This data were consequently correlated to the lower rate of patients (53.5%) treated with induction ChT before the SBRT, unlike the six out of seven prospective studies previously cited, which included induction ChT in the therapeutic program for all analyzed patients. To confirm the role of induction ChT in terms of improved survival, in our study we achieved a statistically significant correlation between induction ChT administered before SBRT and OS (*p* = 0.004) and between ChT after SBRT and OS (*p* = 0.019). These results suggested the crucial role of the patients’ selection and the timing of multimodality treatment in LAPC.

Among the retrospective studies, we mentioned the experiences of the Department of Radiation Oncology in Tampa (Florida) and Baltimore (Maryland) [19]. They performed SBRT in patients affected by BRPC and LAPC after ChT with an RT regimen delivered in five consecutive daily fractions with median total radiation doses of 30 Gy to the tumor and a 40 Gy dose painted to tumor-vessel interfaces. Patients affected by LAPC were 49. The estimated median OS was 15.0 months for LAPC patients. Five of 21 (24%) LAPC patients receiving FOLFIRINOX ChT underwent R0 resection. For those not undergoing resection, one-year locoregional control was 78%, consistent with our local control outcome (81.9%).

Further confirmation of these suggestions may come from a prospective Phase II study involving induction chemotherapy and then SBRT for patients with LAPC (ClinicalTrials.gov Identifier: NCT03158779).

Another crucial issue related to SBRT in pancreatic cancer is the toxicity profile. In 2015, Brunner et al. [40] reviewed the literature on pancreatic SBRT published from 2004 to 2013 and showed that late toxicity G2 and G3 correlated highly with equivalent radiation dose and survival. This review confirmed that late toxicity ≥ G2 was substantial and predominantly gastrointestinal, reflecting the use of partly inadequate dose constraints for the duodenum and gastrointestinal tract organs. Since then, main prospective and retrospective published studies, as reported in Table 1 and Table 2, showed acute ≥ G3 and late ≥ G3 gastrointestinal toxicity less than 10%, according to the results of a published systematic review and pooled analysis [41].

Our report confirmed these results, with a G2 late toxicity rate <1% and no cases of ≥ G3 toxicity. These data were related, on the one hand, to the priority we give to dose constraints for OaRs in the treatment planning phase (Figure 1) and, on the other hand, to the accuracy of treatment delivery with daily CBCT.

Regarding the role of FDG PET-CT, although previous studies have demonstrated its prognostic value in other cancers, its role in PC is not yet clear. In 2014, the team of Herman and Koong analyzed the prognostic utility of FDG PET-CT in patients with LAPC treated with SBRT. In the analysis of 32 patients undergoing induction ChT and SBRT (33 Gy/5 fr), pre-SBRT tumor metabolic volume (*p* = 0.029) and total lesion glycolysis (*p* = 0.038), defined as tumor metabolic volume multiplied by the mean SUV, are potential predictive factors for OS in patients with LAPC [42]. In our study, the prognostic value of FDG PET-CT was not confirmed, but this data may have been conditioned by the limited number of patients (12%) subjected to this procedure during the simulation phase.

Summarizing the available literature about SBRT in unresectable PC, a wide variety of prescription RT doses, fractionations, and chemotherapy regimens were proven. The value of our study is based on the large number of analyzed patients, all treated with homogeneous dose prescription, fractionation scheme and RT technique. Therefore, the results of our study therefore reflect, compared to the data in the literature, our strengths, including above all a high and homogeneous dose. The limits of our analysis are represented by the retrospective nature, the heterogeneity in ChT regimes administered before or after SBRT and unfavorable patients’ selection, particularly in terms of age and comorbidities, which adversely affected the use of an integrated treatment of ChT and RT in about 50% of analyzed cases.

Despite this, in these elderly and frail patients, excluded from ChT and otherwise candidates for best supportive care only, SBRT has proven to be a valid therapeutic option in terms of LC, toxicity, and survival.

## 5. Conclusions

SBRT might represent an effective and safe therapeutic option in the multimodal treatment of patients affected by unresectable LAPC. Local control was sustained, and overall survival has proven to be superior when SBRT was sequentially integrated with chemotherapy. Given the retrospective nature of the study, future prospective studies are needed to identify the best treatment timing and therapeutic strategy in combination with systemic therapy to increase the survival of these patients.

## Figures and Tables

**Table 1:** Prospective studies of SBRT in patients with BRPC/LAPC from 2013 to 2021.

Author	Patients	Clinical Stage	SBRT (Dose/Fraction)	Pre-, Post-SBRT Therapy	mFU (Months)	LC	PFS	OS	Gastrointestinal Toxicity ≥ G3 (%)
Gurka et al., 2013 [11]	11	LAPC	25 Gy/5 fr	Gemcitabine pre + post-SBRT	Na	Na	mPFS: 6.8 months	mOS: 12.2 months	Acute ≥ G3: 0% Late ≥ G3: 0%
Tozzi et al., 2013 [12]	30	LAPC Local Relapse	45 Gy/6 fr	Multiple ChT schemes pre + post-SBRT	11	1y: 77% 2y: 75%	mPFS: 8 months	mOS: 11 months 1y: 47%	Acute ≥ G3: 0% Late ≥ G3: 0%
Herman et al., 2015 [13]	49	LAPC	33 Gy/5 fr	Gemcitabine pre + post-SBRT	13.9	1y: 78%	mPFS: 7.8 months 1y: 32% 2y: 10%	mOS: 13.9 months 1y: 59% 2y: 18%	Acute G3: 10.2%; G4: 2% Late G3: 6.4%; G4: 2%
Comito et al., 2017 [14]	45	LAPC	45 Gy/6 fr	Multiple ChT schemes (71% pre-SBRT and 41% post-SBRT)	13.5	mLC: 26 months 1y: 87% 2y: 87%	mPFS: 8 months 1y: 39% 2y: 15%	mOS: 13 months 1y: 59% 2y: 18%	Acute ≥ G3: 0% Late ≥ G3: 0%
Teriaca et al., 2021 [15]	39	LAPC	40 Gy/5 fr	FOLFIRINOX pre-SBRT (8 cycles) + surgery post-SBRT (14%)	13	mLC: 36.3 months 1y: 81% 3y: 53%	mPFS: 10.7 months 1y: 43% 3y: 15%	mOS: 18 months 1y: 77% 3y: 13%	Acute G3: 10.2%
Chen Zhao et al., 2020 [16]	45	LAPC (20) BRPC (25)	40–62 Gy/5–10 fr	Multiple ChT schemes pre-SBRT + surgery post-SBRT (71.1%)	14.7	1y: 95%	1y: 72% 2y: 58%	mOS: 13.8 months 1y: 67% 2y: 36%	Acute ≥ G3: 0% Late ≥ G3: 0%
Zhu et al., 2021 [17]	63	LAPC	35-40Gy/5 fr	S-1 (6 cycles) post-SBRT	15.8	Na	mPFS: 10.1 months	14.4 months	Acute G3: 14.3% Late G3: 4.8%

Na: not available.

**Table 2:** Retrospective studies of SBRT in patients with BRPC/LAPC from 2013 to 2021.

Author	Patients	Clinical Stage	SBRT (Dose/Fraction)	Pre-, Post-SBRT Therapy	mFU (Months)	LC	PFS	OS	Gastrointestinal Toxicity ≥ G3 (%)
Moningi et al., 2015 [18]	88	LAPC (74) BRPC (14)	25–33 Gy/5 fr	Multiple ChT schemes pre-SBRT (87.5%)	13.1	mLC: 13.9 months 1y: 61% 2y: 14%	mPFS: 9.9 months 1y: 41% 2y: 11%	mOS: 18.4 months 1y: 73% 2y: 24%	Acute G3: 3.4% Late G3: 5.7%
Mellon et al., 2015 [19]	159	LAPC (49) BRPC (110)	20–50 Gy/5 fr	Multiple ChT schemes pre-SBRT + surgery post-SBRT (38%)	14	mLC: 12.7 months 1y: 78%	Na	mOS: 18.1 months	Acute ≥ G3: 7% Late ≥ G3: 7%
Gurka et al., 2017 [20]	38	LAPC (28) BRPC (6) Unresectable for clinical comorbidities (4)	25–30 Gy/5 fr	Multiple ChT schemes pre-SBRT	Na	Na	mPFS: 9.2 months	mOS: 14.3 months	Acute G3: 5.2% Late G3: 5.2%; G4: 2.6%; G5: 2.6%
Zhu et al., 2017 [21]	417	LAPC (218) BRPC (105) Metastatic (94)	30–46.8 Gy/5–8 fr	Multiple ChT schemes pre-SBRT + post-SBRT (11.2%)	11	mLC: 10 months 1y: 26.6%	mPFS: 8 months 1y: 18.2%	mOS: 10 months 1y: 35.5%	Acute G4: 0.5% Late G3: 0%
Mazzola et al., 2018 [22]	33	LAPC	42-45 Gy/6 fr	Multiple ChT schemes pre-SBRT (72%) + post-SBRT (60%) and surgery post-SBRT (18%)	18	1y: 81%	Na	1y: 75%	Acute ≥ G3: 0% Late ≥ G3: 0%
Toesca et al., 2020 [23]	149	LAPC	20–45Gy/3-6 fr	Multiple ChT schemes pre-SBRT	15	1y: 86%	Na	16 months	Acute ≥ G3: 13% Late ≥ G3: 0%
Shen et al., 2020 [24]	56	LAPC	40 Gy (30–50 Gy)/5 fr	GEM-CAP (2 cycles) and concurrent to RT (4 cycles)	17	1y: 85%	mPFS: 12 months 1y: 48.2% 2y: 14.3%	mOS: 19 months 1y: 82.1% 2y: 35.7%	Acute G3: 10.8% Late G3: 5.4%

Na: not available.

**Table 3:** Patients’ and treatment’s characteristics (n = 142).

**Median age (range)**	**71 years (41–91)**
<70 years	63 patients
≥70 years	79 patients
Tumor location (number of patients, %)	
Head	91 (64.1%)
Uncinate process	23 (16.2%)
Body	20 (14.1%)
Tail	3 (2.1%)
Isthmus	5 (3.5%)
CA19.9 before SBRT	124 (87.3%)
<300 U/mL	79 (63.7%)
≥300 U/mL	45 (36.3%)
Not available	18 (12.7%)
Median (range) [U/mL]	102.75 (0.8–12.000)
CA19.9 after SBRT	
Median value (range) [U/mL]	35 (0.4–8594)
Median diameter (cm, range)	3.7 (1.4–9.3)
Median volume (cc, range)	
CTV	31.6 (2.75–187)
PTV	71.3 (17.6–321)
Chemotherapy before SBRT	
Yes	76 (53.5%)
No	66 (46.5%)
Chemotherapy scheme before SBRT	
Gemcitabine	7 (9.2%)
FOLFIRINOX	18 (23.7%)
Gemcitabine + nab-paclitaxel	17 (22.3%)
GEMOX	21 (27.6%)
PEX-G	10 (13.2%)
Others	3 (4%)
Chemotherapy after SBRT	
Yes	42 (29.6%)
No	100 (70.4%)
Chemotherapy scheme after SBRT	
Capecitabine-based	5 (12%)
FOLFIRINOX	6 (14.3%)
Gemcitabine-based	22 (52.4%)
Irinotecan	2 (4.7%)
Others	7 (16.6%)

FOLFIRINOX: folinic acid, fluorouracil, irinotecan, oxaliplatin; GEMOX: gemcitabine, oxaliplatin; PEX-G: cisplatin, epirubicin, 5-fluorouracil, gemcitabine.

**Table 4:** Response to treatment according to RECIST criteria.

Tumor Response	Number of Patients (%)
Complete Response	1 (0.7%)
Partial Response	31 (22%)
Stable Disease	81 (57%)
Progression Disease	29 (20.3%)

**Table 5:** Univariate analysis.

	Local Control			Overall Survival		
	HR	95%CI	*p* Value	HR	95%CI	*p* Value
Age > 70 years	1.11	0.53–2.32	0.776	1.46	1.02–2.08	0.037
Tumor Diameter	0.98	0.95–1.01	0.346	1.00	0.99–1.02	0.400
CTV Volume	0.99	0.98–1.01	0.881	1.00	0.99–1.01	0.163
PTV Volume	0.99	0.99–1.00	0.865	1.00	0.99–1.00	0.075
Time from diagnosis to SBRT	0.97	0.91–1.04	0.487	0.98	0.96–1.01	0.460
CA19-9 before SBRT	1.00	0.99–1.00	0.740	1.00	0.99–1.00	0.417
CA19-9 after SBRT	-	-	-	-	-	-
ChT before SBRT	0.85	0.40–1.79	0.675	0.59	0.41–0.84	**0.004**
ChT after SBRT	1.71	0.81–3.57	0.155	0.63	0.43–0.92	**0.019**
Local progression	-	-	-	0.90	0.60–1.37	0.655
Distant progression	1.10	0.26–4.70	0.890	0.89	0.45–1.77	0.758

## Data Availability

Not applicable.

## Supplementary Materials

The following supporting information can be downloaded at: https://www.mdpi.com/article/10.3390/curroncol30070513/s1, Table S1: Multivariable Analysis.
