# Supplementary material for: Can STEreotactic Body Radiation Therapy (SBRT) Improve the Prognosis of Unresectable Locally Advanced Pancreatic Cancer? Long-Term Clinical Outcomes, Toxicity and Prognostic Factors on 142 Patients (STEP Study)
Source: Curr Oncol. 2023 Jul 24;30(7):7073–88. doi: 10.3390/curroncol30070513 (PMC10378012; doi:10.3390/curroncol30070513)
Supplement: Supplementary file 1 [file curroncol-30-00513-s001.zip › curroncol-2453440-supplementary.pdf]

Table S1. Multivariable analysis									
	HR	95%CI	P value	HR	95%CI	P value	HR	95%CI	
Age > 70y	1.17	0.77 – 1.78	0.447	-	-	-	-	-	-
CT before SBRT	0.73	0.46 – 1.15	0.183	-	-	-	-	-	-
CT after SBRT	0.76	0.49 – 1.19	0.239	-	-	-	-	-	-
